# Far Beyond Declarative and Non-Declarative Memories

**DOI:** 10.3389/fnbeh.2014.00173

**Published:** 2014-05-08

**Authors:** Ekrem Dere, Armin Zlomuzica

**Affiliations:** ^1^UFR des Sciences de la Vie (UFR 927), Université Pierre et Marie Curie, Paris, France; ^2^Max Planck Institute of Experimental Medicine, Göttingen, Germany; ^3^Mental Health Research and Treatment Center, Ruhr University Bochum, Bochum, Germany

**Keywords:** consciousness, concept formation, episodic memory, awareness, declarative memory, nondeclarative memory

According to general learning theory two major forms of learning can be observed across species. These universal forms of learning are classical and operant conditioning (Tarpy, 1997). Although both forms of conditioning can occur in the absence of awareness of the contingencies between CS (conditioned stimulus) and UCS (unconditioned stimulus) (or action – consequence contingencies) (Huston and Oitzl, 1989; Clark and Squire, 1998), close temporal proximity between a CS and a UCS is required to form an association. In other words, long-term association between a CS and a UCS (or an operant and its consequence) requires two distinct events to occur simultaneously or with little delay (in the range of ms) between them.

Now let us consider the following question: Can two events be associated or integrated into a linked mnemonic representation even if they happen at different times (with a delay of more than 1 min between the two events) and if one of these events is not detected consciously? The conventional answer would be “certainly not.” However, the research article “Integrating events across levels of consciousness” by Henke et al. (2013) demonstrates that this is indeed possible by showing that two temporally distant events that have plainly generated memories both conscious and unconscious were integrated mentally into a linked mnemonic representation.

In Henke et al.’s (2013) study participants sequentially viewed various unrelated word-pairs such as “tree desk.” Half of these pairs shared one member, for example “tree desk” and “desk fish” to promote event integration. The experimental procedure varied the delay between the presentations of the two word-pairs with a shared member in a way that would preclude simple forms of latent learning due to classical conditioning. Moreover, one word pair was presented subliminally (invisible) while the other word pair was presented supraliminally (visible). Nevertheless participants integrated the two word-pairs (events) into a linked mnemonic representation by judging the word pair that shared one member as semantically closer than the word-pairs that did not shared a member. This judgment of semantic relatedness was evident even if the two word-pairs were temporally distant, i.e., presented at different times. Henke et al. (2013) conclude that this finding suggests that: “connections between mental representations or between nodes in the semantic network, which have been co-activated in the same encoding context, acquire a greater linkage strength leading to the impression of stronger conceptual relatedness.”

This interpretation has important implications for the question of how we generate our knowledge and concepts about the world. Much of that knowledge results from integrating memories for specific events or experiences into semantic concepts (Squire and Zola, 1998). For example, our concept of a “restaurant visit” consists of a sequence or chain of events and actions one must perform within a particular setting to qualify it as a “restaurant visit.” These single sequences have been distilled from the integrated memories of many events that happened at different times and that can be roughly classified as “restaurant visits,” including visits, e.g., to fast food and sushi restaurants. However, the mechanisms underlying this type of concept formation are still unknown. Given that no explicit intention exists to form a concept or to integrate similar events that happened at different times into a semantic concept, this process must be initiated unconsciously possibly due to a hidden concept formation algorithm that detects similarities between events that happened at different times and places.

The findings show that conscious and unconscious memories for events that happened at different times can join together into a mnemonic representation. The work by Henke et al. (2013) further suggest that people possess learning capabilities that do not fall under the categories of declarative and non-declarative memory systems (Squire, 2004). These new findings imply that people might have at least one qualitatively different learning mechanism that cannot be classified within this categorical dichotomy. The results by Henke et al. reveal a learning process not dependent on consciousness (declarative memory) and that violates the principle that a close spatial and temporal contiguity is essential for classical conditioning (non-declarative memory).

In future studies, it would be worth to determine the maximum temporal delay between supra- and subliminally presented word-pairs, that would still allow an association between those word-pairs, that share a member, but have been presented at different times. Another interesting question to ask would be to test whether the maximum temporal delay could be extended by using shared member words that have an emotional valence or would induce an emotional arousal. If it turns out that the delay between the presentation of word-pairs can be extended to several minutes or even up to hours, such a finding would indicate that the association formed is not exclusively due to a priming phenomenon as it has been suggested by the authors.

An interesting direction for future studies would be the examination of neurobiological underpinnings of this unique type of learning. These studies could broaden our knowledge on the fascinating interplay between our conscious and unconscious memories and mental lives.

The findings by Henke et al. might also have implications for the concept of episodic memory that is thought to be part of the declarative memory system. Episodic memories are extremely vivid and detailed memories for specific events and their spatial–temporal context (Dere et al., 2010; Pause et al., 2013). We have postulated that episodic memories are generated without an explicit intention to do so (automatically or unconsciously) through a learning mechanism that resembles classical conditioning triggered by events that are emotionally arousing. Furthermore, we have postulated that the temporal context of an event is constructed by relating or associating the event to episodic memories (so-called “anchor events”) that have been generated earlier and to episodic memories that will be generated in the future. In this way, the temporal context of an event is reconstructed and stored as temporal order information, e.g., event *B* happened after event *A* but prior to event *C* (Dere et al., 2004, 2008, 2010; Pause et al., 2013; Zlomuzica et al., 2014). The findings by Henke et al. seem to support our concept of episodic memory formation by showing that the strict distinction between declarative and non-declarative memories might not account for all forms of learning and memory, including episodic memories and that an association between temporally distant events in terms of temporal order relationships can indeed be formed automatically/unconsciously.

## Conflict of Interest Statement

The authors declare that the research was conducted in the absence of any commercial or financial relationships that could be construed as a potential conflict of interest.
